# Prevention of respiratory syncytial virus infection with probiotic lactic acid bacterium *Lactobacillus gasseri* SBT2055

**DOI:** 10.1038/s41598-019-39602-7

**Published:** 2019-03-18

**Authors:** Kei Eguchi, Naoki Fujitani, Hisako Nakagawa, Tadaaki Miyazaki

**Affiliations:** 1 Milk Science Research Institute, Megmilk Snow Brand Co., Ltd., 1-2, Minamidai, 1-chome, Kawagoe, Saitama, 350-1165 Japan; 2 Department of Probiotics Immunology, Institute for Genetic Medicine, Hokkaido University, N15W7, Kita-ku, Sapporo, 060-0815 Japan

## Abstract

*Lactobacillus gasseri* SBT2055 (LG2055) is a probiotic lactic acid bacterium with multifunctional effects, including the prevention of influenza A virus infection in mice, reduction of adipocyte size in mice, and increased lifespan in *C. elegans*. We investigated whether LG2055 exhibits antiviral activity against respiratory syncytial virus (RSV), a global pathogen for which a preventive strategy is required. Following oral administration of LG2055 in mice, the RSV titre in the lung was significantly decreased, while body weight was not decreased after virus infection. Additionally, the elevated expression of pro-inflammatory cytokines in the lung upon RSV infection decreased after LG2055 administration. Moreover, interferon and interferon stimulated genes were upregulated by LG2055 treatment. Comparative cellular proteomic analysis revealed that SWI2/SNF2-related CREB-binding protein activator protein (SRCAP) was a candidate for the antiviral activity of LG2055 against RSV. There was a positive correlation between the inhibition of RSV replication and the suppression of SRCAP expression and RSV replication was suppressed by SRCAP silencing. Since SRCAP is a scaffold protein to which viral non-structural proteins bind, the downregulation of SRCAP induced by LG2055 could provide new insights about the inhibition of RSV replication. In summary, our study demonstrated that LG2055 has prophylactic potential against RSV infection.

## Introduction

Human respiratory syncytial virus (RSV), an enveloped negative-sense RNA virus belonging to the *Paramyxoviridae* family, is the major causative virus of bronchiolitis and pneumonia in children^1,2^. Most children have been infected with RSV by 2 years of age, and between 66,000 and 239,000 children younger than 5 years old are estimated to die every year due to RSV lower respiratory tract infection^1–3^. In addition to children, elderly and immunocompromised individuals are potentially at high risk of becoming severely symptomatic due to RSV infection^4–6^. While the symptoms of RSV infection in healthy adults are relatively milder than those in children and the elderly, the prevention of RSV infection is important, as repeated infections may occur throughout one’s lifetime^7^. Therefore, because RSV-infected adults without severe symptoms could become spreaders of RSV to high-risk individuals, the control of RSV transmission is an important public health issue. Incorporation of therapeutic factors in food could be a means of providing a preventive medicine. The development of vaccines and therapeutic agents against RSV has progressed^6,8^. However, there is no vaccine that has been put to practical use, and specific therapeutic agents against RSV infection are limited to the bench, but not yet the bedside, underscoring the need for new prophylactic and therapeutic strategies for RSV infection.

Probiotics are beneficial live microorganisms that confer a benefit to the host. In recent years, they have attracted research attention, as they are able to promote human health when ingested in adequate amounts^9^. *Lactobacillus* species are the most representative probiotics, and their various physiological and immunological effects have been reported. In particular, the effects of *Lactobacillus* species against various infectious diseases, including influenza, have been actively investigated^10–13^. For example, it was reported that the activity of natural killer T-cells was upregulated by *Lactobacillus acidophilus* L-92, resulting in prevention of influenza virus infection in mice^14^. In addition, the cell wall of *Lactobacillus acidophilus* NCFM was found to upregulate anti-viral gene expression via the Toll-like receptor pathway in mice^15^.

*Lactobacillus gasseri* SBT2055 (LG2055), was originally isolated from human feces^16^, and has been described as a multifunctional probiotic. The resistance of LG2055 to bile acid is a property that facilitates its colonization in the human gut, enabling it to contribute to the physiological improvement of the intestinal environment^17,18^. This probiotic-mediated improvement, in turn, promotes various biological effects, such as prevention of abdominal obesity in humans and rats^19,20^, extension of lifespan in *Caenorhabditis elegans*^21^, promotion of IgA production in mice^22^, and prevention of influenza A virus infection in mice^10^.

We herein present the antiviral effect of LG2055 against RSV in addition to the above beneficial functions of LG2055, demonstrating that LG2055 suppresses the replication of RSV *in vitro* and i*n vivo*. Furthermore, to elucidate the molecular mechanism by which LG2055 exerts antiviral activity against RSV, we performed a comparative proteomic analysis of host cells using nanoLC-ESI-MS/MS. As a result, SWI2/SNF2-related CREB-binding protein activator protein (SRCAP) was found to be involved in the RSV proliferation inhibitory effect of LG2055. Based on our findings, we propose that LG2055 is a promising probiotic useful for the prevention of RSV infection and alleviation of associated symptoms.

## Results

### Inhibitory effect of LG2055 on RSV replication *in vitro*

**Figure 1:**
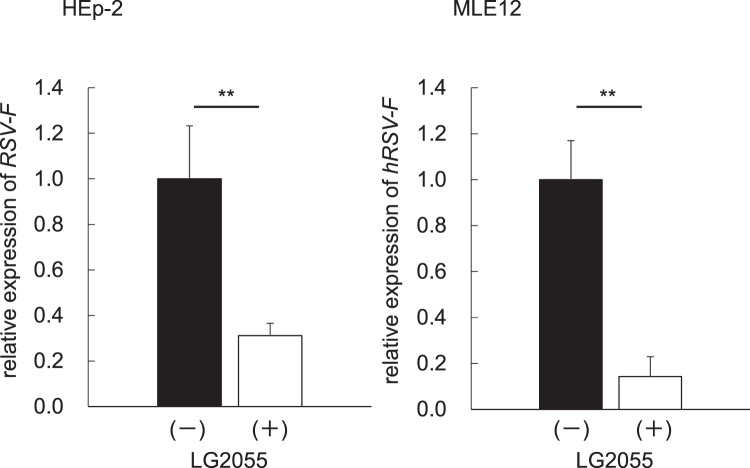
LG2055 protected human and mouse cells from RSV infection *in vitro*. Human and mouse epithelial cells, HEp-2 and MLE12, respectively, were treated with or without LG2055 (50 μg/ml) for 24 h and infected with RSV (0.00067 TCID_50_/cell). After 48 h postinfection, RSV-specific gene expression in cells was assessed by real-time qPCR. Data are presented as means + SEM. ^**^P < 0.01 (Two-tailed Student’s *t* test).

To evaluate the antiviral activity of LG2055 against RSV, we performed an infection assay for RSV strain A2 in HEp-2 human laryngeal epithelial cells and MLE12 mouse lung epithelial cells. Prior to virus infection, cells were pretreated with lyophilized LG2055 at a final concentration of 50 μg/ml for 24 h, and then infected with RSV at 0.00067 TCID_50_/cell for 48 h. The addition of lyophilized LG2055 without RSV infection did not affect cell viability (Supplement Fig. 2). The expression level of the virus-specific gene coding F-protein (*RSV-F*), critical for the fusion to host cells, was analysed from total RNA obtained from harvested cells. As shown in Fig. 1, the expression of *RSV-F* was significantly suppressed in LG2055-treated HEp-2 and MLE12 cells, indicating that LG2055 inhibited RSV replication *in vitro*.

### Inhibitory effect of LG2055 on RSV replication *in vivo*

**Figure 2:**
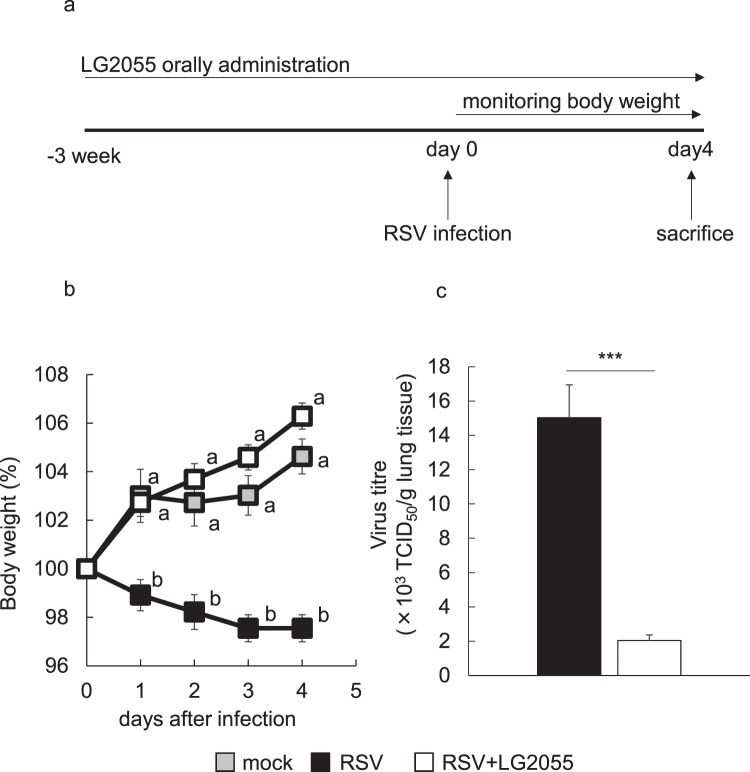
Orally administered LG2055 protected mice from RSV infection. BALB/cCrSlc mice were orally administered LG2055 (2 × 10^9^ cfu/head) or 25% trehalose solution once a day for 21 d. After administration, mice were intranasally infected with the RSV A2 strain (5 × 10^6^ TCID_50_/head). Oral administration continued until day 4 postinfection. Mock: orally administered 25% trehalose solution and RSV uninfected (n = 5), RSV: orally administered 25% trehalose solution and RSV infected (n = 10), RSV + LG2055: orally administered LG2055 and RSV infected (n = 10). (a) Feeding and infection protocols. (b) Body weight (%) compared to day 0 (100%). Data are presented as means ± SEM. Means with the same letter do not differ (*P* ≥ 0.05, Tukey-kramer test). (c) Virus titres in mouse lung homogenates. Virus titres were measured at day 4 postinfection. Data are presented as means + SEM. ^***^P < 0.001 (Two-tailed Student’s *t* test).

To investigate whether the antiviral activity and prophylactic effects of LG2055 against RSV infection also occurred *in vivo*, LG2055 was orally administered to mice daily for 21 d (2 × 10^9^ cfu/mouse/day in 25% trehalose solution), and the mice were then infected with RSV at 5 × 10^6^ TCID_50_ (day 0), as shown in Fig. 2a. Oral administration of LG2055 and monitoring of body weight were performed daily until sacrifice on day 4 after virus infection.

The body weight of RSV-infected mice was not decreased by LG2055 administration compared with uninfected control mice, while that of RSV-infected mice without LG2055 administration was significantly decreased, as shown in Fig. 2b. The virus titre in lung tissue homogenates was significantly suppressed by LG2055 administration at day 4 postinfection (Fig. 2c). These data indicated that continuous ingestion of LG2055 prior to RSV infection effectively inhibited RSV replication and the severity of associated symptoms.

**Figure 3:**
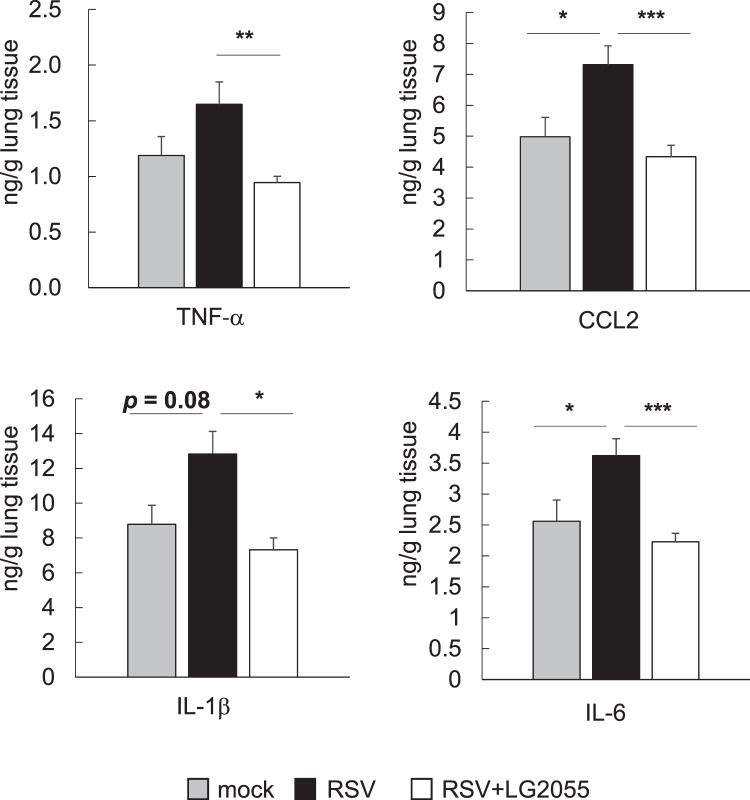
Orally administered LG2055 reduced proinflammatory cytokine production in the lung resulting from RSV infection. Mice were orally administered LG2055 (2 × 10^9^ cfu/head/day) or a 25% trehalose solution for 21 d, and were then infected with RSV or not infected (mock: n = 5, RSV: n = 10 and RSV + LG2055: n = 10, similar to Fig. 2). The concentration of TNF-α, CCL2, IL-1β, and IL-6 in lung homogenates was measured using ELISA. Data are presented as means + SEM. ^*^P < 0.05, ^**^P < 0.01 and ^***^P < 0.001 (Dunnett’s test, vs. RSV).

Based on the result that oral LG2055 administration resulted in the prevention of RSV infection, we hypothesized that LG2055 could function by reducing the inflammatory response in the lungs of RSV-infected mice. Thus, the expression level of the proinflammatory cytokines TNF-α, CCL2, IL-1β, and IL-6 in lung tissue homogenates was evaluated, as these factors are known to be elevated by RSV infection^23–26^. Although the expression levels of all the measured cytokines tended to be upregulated following infection with RSV, those of LG2055-treated mice were equivalent to those in RSV-uninfected healthy mice at day 4 after RSV infection (Fig. 3). Except for IL-6, the expression of proinflammatory cytokines in LG2055-treated mice was already suppressed at day 1 after virus challenge, and the expression of IL-6 resembled that in uninfected mice at day 4 after virus challenge (Supplement Fig. 1). These results indicated that oral administration of LG2055 inhibited the inflammatory response in the lungs of mice after RSV infection.

**Figure 4:**
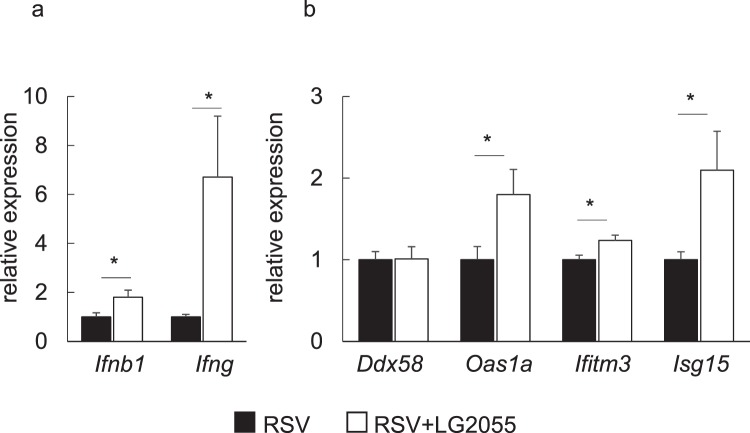
Orally administered LG2055 upregulated antiviral gene expression in the mouse lung. Mice were orally administered LG2055 (2 × 10^9^ cfu/head/day) or a 25% trehalose solution for 21 d, and were then infected with RSV or not infected (mock: n = 5, RSV: n = 10 and RSV + LG2055: n = 10, similar to Fig. 2). Gene expression in lung homogenates at day 4 postinfection was assessed by real-time PCR analysis. (**a**) Gene expression of type I and type II interferon. (**b**) Gene expression of interferon-stimulated genes. Data are presented as means + SEM. ^*^P < 0.05, ^**^P < 0.01 (Two-tailed Student’s t test).

Our previous study reported that LG2055 treatment alone did not upregulate type I IFN in mouse lungs^10^. We next evaluated whether LG2055 could activate an antiviral type I and II IFN response *in vivo* during RSV infection, as both type I and II IFN effectively inhibit RSV infection^27,28^. Oral administration of LG2055 increased the expression of IFN-β and IFN-γ at the mRNA level in the mouse lung. The expression of IFN-β was sharply elevated one day after RSV infection, and the expression amount of IFN-β tended to be higher in LG2055-administered mice than in untreated mice (Supplement Fig. 3) at day 1. By day 4 postinfection, IFN-β decreased to approximately one-fifth of the first day (Fig. 4a).

Furthermore, the mRNA level of OAS1a and ISG15 was also increased in lung tissue homogenates of mice treated with LG2055 at day 4 after RSV infection (Fig. 4b). OAS1a, ISG15 and IFITM3 play an important role in the protection of epithelial cells from RSV infection^27,29,30^. Our results indicated that oral administration of LG2055 induced an antiviral state against RSV infection in the lungs of mice. However, the gene expression level of *Ddx58* (RIG-I) involving in the antiviral response^31^ was found to be no significant difference between LG2055-fed and control mice during RSV infection (Fig. 4b).

### Proteomic analysis for discovering essential proteins involved in the inhibition of RSV replication

**Figure 5:**
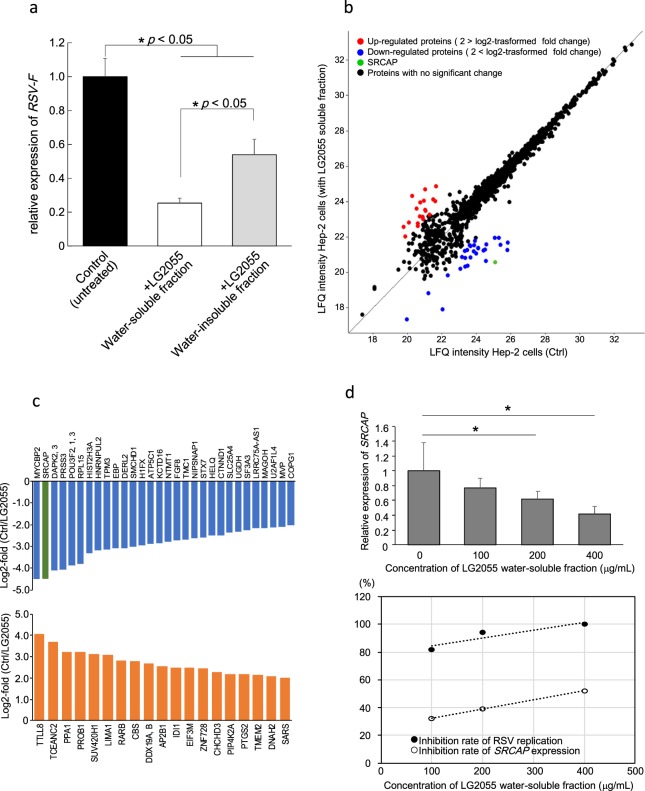
Comparative proteomic analysis of HEp-2 cells treated with LG2055. (**a**) Antiviral activity of water-soluble and water-insoluble components fractionated from LG2055. (**b**) Scatterplot showing the protein expression change between LG2055 soluble fraction-treated and untreated HEp-2 cells. Proteins with a significant change with log2-fold change > 2 (upregulated) and < 2 (downregulated) are represented by red and blue lines, respectively; SRCAP is indicated in green. Proteins with no significant change by the addition of LG2055 are indicated in black. (**c**) Bar plot of the log2-transformed fold changes (LG2055+/untreated) of the downregulated or upregulated proteins in LG2055-treated HEp-2 cells (top and bottom, respectively). Listed proteins are indicated by their gene name. SRCAP is highlighted in green. (**d**) Correlation between the inhibition rate of RSV replication and the inhibition rate of SRCAP expression by treatment with the LG2055 water-soluble fraction. Data are presented as means + SD. ^*^P < 0.05 (two-tailed Student’s *t* test).

We hypothesized that the components of LG2055 assimilated from the intestine could also exhibit antiviral activity by directly acting on respiratory epithelial cells. In the process of determining the active substances from probiotic lactic acid bacterium, the active molecules are identified by dividing into a water soluble fraction and an insoluble fraction depending on the physical properties. For example, the peptide from a lactic acid bacterium *Enterococcus mundtii* ST4V recovered from water-soluble fraction suppressed Herpes simplex virus type I, type II and measles virus^32^. On the other hand, nasally administering peptidoglycans of a certain lactic acid bacterium *Lactobacillus rhamnosus* CRL1505 collected from water-insoluble fraction suppressed infection of respiratory syncytial virus^33^. Thus, to identify factors with antiviral activity, we first fractionated lyophilized LG2055 into water-soluble and water-insoluble fractions and examined the anti-RSV activity of each fraction. While both fractions suppressed the replication of RSV, the water-soluble fraction showed an approximately 2.1-fold stronger antiviral activity than the insoluble fraction (Fig. 5a).

Next, to elucidate the molecular mechanism by which the water-soluble fraction of LG2055 mediates the alteration of protein expression and exerts antiviral activity against RSV, we performed fast proteomic screening. We performed a comparative shotgun proteomic analysis of HEp-2 cells with either the presence or absence of the water-soluble fraction of LG2055. Tryptic-digested peptides were subjected to mass spectrometry (MS) and a label-free quantitation (LFQ) procedure was applied to analyse the alteration of the protein expression pattern according to LG2055 fraction exposure.

Proteomic analysis based on MS led to the identification of a total of 1120 proteins. We defined a 4-fold change in protein expression (more than 2 in log2-transformed fold change) by the addition of the LG2055 water-soluble fraction as a significant change. According to this criterion, 29 downregulated and 19 upregulated proteins were identified. A scatterplot showing the alteration in protein expression levels, and the upregulated and downregulated proteins displaying >2.0 and <−2.0 of log2-transformed fold change with addition of the LG2055 water-soluble fraction are shown in Fig. 5b,c and Table S2. The top 5 upregulated proteins were TTLL8, TCEANC2, PPA1, PROB1, and SUV420H1, while the top 5 downregulated proteins were MYCBP2, SRCAP, DAPK2 or 3, POU3F2 or 1 or 3, and RPL15. Among all detected proteins, we extracted a promising candidate protein that was expected to be critical for the regulation of virus replication. SWI2/SNF2-related CREB-binding protein activator protein (SRCAP) was one of the most downregulated proteins due to supplementation of the LG2055 water-soluble fraction, showing an approximately 22-fold decrease in protein amount compared with that in control HEp-2 cells without LG2055 exposure. SRCAP has been reported to potentially have the ability to interact with viral non-structural (NS) protein^34,35^. Since RSV possesses NS, a change in the protein expression level of SRCAP could potentially affect the replication of RSV in host cells.

To determine whether the addition of LG2055 soluble fraction regulates SRCAP expression at the gene level, and whether there is a correlation between the fluctuation of SRCAP expression and inhibition of RSV replication, we analysed the alteration of *SRCAP* expression in HEp-2 cells with or without exposure to the LG2055 water-soluble fraction. *SRCAP* expression was decreased with LG2055 fraction exposure in a dose-dependent manner. That is, as the concentration of LG2055 water-soluble fraction increased, the expression level of *SRCAP* decreased (Fig. 5d). When the soluble fraction of LG2055 was added to HEp-2 cells at a final concentration of 100, 200 and 400 μg/ml, the expression level of *SRCAP* was suppressed by approximately 32.8%, 38.1% and 52.1%, respectively. Next, in RSV infection experiments, addition of the LG2055 fraction at a final concentration of 100, 200, and 400 μg/ml resulted in decrease of 82.0%, 94.0%, and 99.6%, respectively, in the expression of *RSV-F* in RSV-infected cells. As shown in Fig. 5d, there was a distinct correlation between the *SRCAP* expression level and the rate of RSV replication, suggesting that suppression of SRCAP could be a major mechanism by which LG2055 inhibits RSV replication, and that LG2055-mediated inhibition of SRCAP expression is an effective probiotic-related strategy to suppress virus replication.

Finally, to investigate whether the inhibition of SRCAP directly impacts RSV replication, the silencing of SRCAP in Hep-2 cells using siRNA was performed. It was revealed that the expression of RSV F-protein was significantly suppressed by silencing of SRCAP, suggesting that SRCAP was one of the essential factors for RSV replication.

## Discussion

We demonstrated that LG2055 inhibited RSV replication *in vitro* and *in vivo*. RSV is a common respiratory virus, but despite the development of vaccines and specific therapeutic agents against RSV^6,8^, no vaccine or specific therapeutic agent is currently used clinically. In particular, individuals with weak immunity are at risk of presenting with severe symptoms due to RSV infection. Therefore, effective preventive strategies against RSV are required.

Oral administration of LG2055 suppressed the inflammatory response in the lungs of mice during RSV infection, as the protein levels of the pro-inflammatory cytokines IL-6, TNF-α, IL-1β, and CCL2 were retained at the equivalent levels to those of control mice. LG2055 could contribute to the alleviation of allergic airway disease induced by RSV infection, as TNF-α has been reported to play a critical role in the exacerbation of airway disease during RSV infection^25^.

It has been demonstrated in several studies that type I and II IFN protect mice from RSV infection^27,28^. Our results showed that LG2055 enhanced IFN-β and IFN-γ expression at the gene level in the lungs of mice after RSV infection, consistent with data from previous studies showing that several lactic acid bacteria induce IFN-β or IFN-γ in the lungs of mice infected with respiratory virus to contribute to virus clearance^15,36,37^.

The expression of OAS1a and ISG15, factors that protect epithelial cells from RSV infection^27,29^, were also increased at the gene level with LG2055 administration, further supporting the notion that orally-administered LG2055 has a potential role in the prevention of RSV infection. On the other hand, our previous study showed that LG2055 upregulated *Oas1a* expression in the lung regardless of influenza virus infection^10^. Thus, the induction of *Oas1a* expression by LG2055 could contribute to antiviral activity against RSV. Further, the finding that enhanced *Oas1a* expression with LG2055 administration was still maintained after RSV infection indicated that the induction of *Oas1a* expression was not inhibited by RSV. It has been reported that RIG-I (*Ddx58*), an important viral sensor during the RSV infection stage, is involved in the antiviral response when alveolar macrophages in mice detect RSV^31^. However, our results showed that LG2055 did not elevate *Ddx58* expression in RSV infection, suggesting that the inhibitory effect of LG2055 on RSV replication was not mediated by RIG-I induction. Although IFITM3 expression, which exerts a preventive effect on RSV infection in mice^30^, was slightly increased at the gene level by LG2055 administration, the rate of change was less significant than that of the other evaluated genes, suggesting that induction of this gene was not the main cause of the inhibitory effect on RSV.

**Figure 6:**
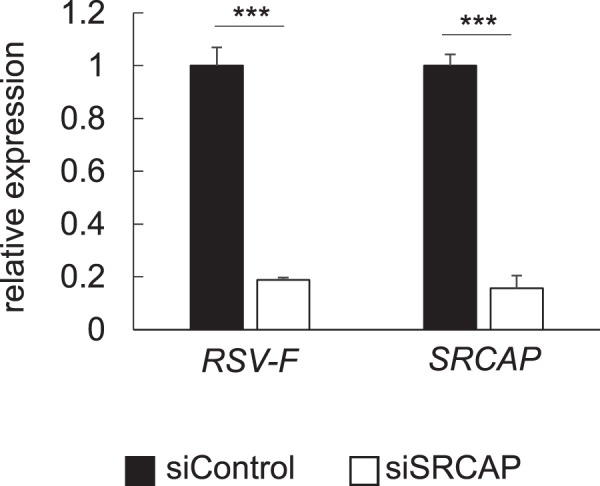
Suppression of RSV replication by silencing of SRCAP using siRNA. (**a**) Silencing efficiency of SRCAP using siRNA compared to control siRNA. (**b**) Expression level of RSV F-protein in SRCAP silenced Hep-2 cells. Data are presented as means + SD. ^***^P < 0.001 (Two-tailed Student’s *t* test).

We additionally performed a proteomic analysis to determine the antiviral mechanism induced by LG2055. We identified SRCAP, one of the most strongly LG2055-downregulated proteins, based on previous findings indicating that SRCAP expression was affected by virus-derived proteins^34^. While SRCAP was originally identified as an activator of CREB-binding protein involved in the enhancement of transcriptional activity^38^, previous studies revealed that SRCAP interacts with NS of the hepatitis C virus to mediate transcription^34,35^. Of particular note is that SRCAP is potentially a scaffold protein for the recognition of viral NS; thus, inhibition of SRCAP expression could result in suppression of the replication of viruses with NS. Indeed, silencing SRCAP expression in Hep-2 cells with siRNA showed significant suppression of RSV replication (Fig. 6), suggesting that SRCAP is one of the essential molecules contributing to RSV replication. To our knowledge, there is no report on substances enabling the downregulation of the expression of SRCAP, except for genetic methods such as siRNA or shRNA treatment.

This study shows that ingestion of probiotic LG2055 will be a promising strategy for the prevention of RSV infection. This was based on two independent mechanisms that induce LG2055 to alter cytokine levels as demonstrated in *in vivo* assays and to downregulate SRCAP expression as seen in *in vitro* assays with epithelial cells. The former is supported previous studies revealing that *Lactobacillus* treatment contributed to the immune function including changes cytokine levels in lung^39,40^. The latter mechanism related to SRCAP contributing to the chromatin remodelling and to deposition of histone H2A.Z at promoter^41^ remains elusive. However, recent study reported that regulation of SRCAP remodelling activity by PCI domain-containing protein 2 contributed to the lymphoid lineage commitment that is an important process in haematopoiesis^42^. Because lymphoid lineage commitment establishes the immune system for host defence against pathogen invasion^42^, from the immunological aspect, SRCAP may be closely related to RSV infection as well as interaction with viral NS protein.

Probiotic lactobacilli, including LG2055 used in this study, are safe to eat and are thought to be effective in alleviating the symptoms associated with RSV infection. However, as a remaining issue to be solved, a limitation of our study was the inability to determine the essential molecules contained within LG2055 required to downregulate SRCAP and inhibit RSV replication. Additionally, the detailed molecular mechanisms by which SRCAP inhibits viral replication must be elucidated.

In summary, our study showed that LG2055 has prophylactic potential against RSV infection. We propose that a structural characterization of the active ingredient contained in LG2055 that enables a decrease in the expression of SRCAP and inhibition of RSV replication is an important future research direction.

## Experimental Procedures

### Lactobacillus gasseri SBT2055 (LG2055) preparation and growth conditions

LG2055 was provided from the Milk Science Research Institute, Megmilk Snow Brand Co., Ltd (Tokyo, Japan). LG2055 was cultured in MRS broth (Becton, Dickinson and Company, East Rutherford, NJ, USA) at 37 °C for 18 h (medium of 1.5 × 10^9^ cfu/ml) and centrifuged at 4 °C for 10 min at 8,000 × *g*. Then, harvested cells were washed twice with sterile PBS and resuspended in 25% trehalose solution (1 × 10^10^ cfu/ml), and suspensions were stored at −80 °C until used for *in vivo* experiments. The rest of the harvested cells were resuspended in distilled water and lyophilized. This lyophilized powder was resuspended in sterile PBS (10 mg/ml) and heat-killed (80 °C, 30 min), then stored at −20 °C until used for *in vitro* experiments.

### Preparation and titration of RSV solution

The RSV A2 strain (RSV) and the human laryngeal carcinoma epithelial cell line HEp-2 were kindly provided by Prof. Hiroyuki Tsutsumi (Sapporo Medical University, Sapporo, Japan). HEp-2 cells were cultured at 37 °C in 5% CO_2_ in DMEM (Sigma-Aldrich Co., St. Louis, MO, USA) supplemented with 5% foetal bovine serum (FBS; Thermo Fisher Scientific, Waltham, MA, USA) and 100 U/ml penicillin-streptomycin (Sigma-Aldrich Co.). For preparation of RSV solution, HEp-2 cells were infected with RSV in serum-free DMEM. At 72 h post infection, cells were scraped and the flask content was pooled, then centrifuged at 410 × g for 10 min to remove cell debris. Virus titres of culture supernatant were measured based on 50% tissue culture infective dose (TCID50) and the supernatant was used for infective experiments.

### *In vitro* RSV infection

HEp-2 cells and the mouse lung epithelial cell-line MLE12 (purchased from ATCC) were prepared in 24-well dishes by seeding 1.5 × 10^5^ cells/well in 0.5 ml of DMEM supplemented with 5% of FBS and cultured overnight at 37 °C. Then, cells were pre-treated with LG2055 by addition of LG2055 suspension to each well at a final concentration of 50 μg/ml and incubated for 24 h. After incubation, the culture medium was removed, and each well was washed with PBS and 0.5 ml of serum-free DMEM containing RSV (0.00067 TCID_50_/ml) and LG2055 (final concentration was 50 μg/ml) were added to each well. After incubation for 48 h, the culture medium was removed, and each well was washed with PBS and 0.5 ml of TRIzol reagent (Thermo Fisher Scientific) was added to extract total RNA.

### *In vivo* RSV infection

BALB/cCrSlc mice (5–7-week-old female, Japan SLC Inc. Shizuoka, Japan) were used in the study. All experiments with mice were conducted under the condition of biosafety level 2. Our animal experiments were approved by the Animal Care and Use Committee of Hokkaido University (approval number; 15–0156) and performed according to the guidelines of the Bioscience Committee of Hokkaido University.

Mice were orally administered LG2055 (2 × 10^9^ cfu/mouse/day, 0.2 ml/mouse/day) or 25% trehalose solution with a ball-type feeding needle for 21 d. After daily administration of LG2055 for 21 d, mice were intranasally infected with RSV (5 × 10^6^TCID_50_/mouse) under anesthetization with isoflurane (Dainippon Pharmaceutical, Osaka, Japan) (day 0).

Daily oral administration of LG2055 or trehalose solution was continued for 4 d after RSV infection. At day 4, mice were sacrificed, and intact lung tissues were collected and snap frozen in liquid nitrogen and stored at −80 °C until further use. The daily body weights were monitored in mice sacrificed at day 4.

### Virus titration and cytokine detection in lung

Lung tissues in 0.5 ml DMEM were homogenized using BEADS CRUSHER μT-12 (TIETECH Co., Aichi, Japan) at 3,000 rpm for 1 min at 4 °C. Homogenates were centrifuged at 800 × *g* for 5 min at 4 °C to collect the supernatant, and virus titres were measured by TCID_50_. To measure cytokine levels in the lung, lung tissue was homogenized using CelLytic MT Cell Lysis Reagent for mammalian tissues (Sigma-Aldrich Co.) containing complete mini protease inhibitors (Roche Diagnostics, Basel, Switzerland) and centrifuged at 4 °C for 10 min at 8,000 × *g*. Cytokine levels in the supernatant of lung tissue homogenates were measured using ELISA kits (BioLegend, San Diego, CA, USA) according to the manufacturer’s instructions.

### Quantitative RT-PCR analysis

Total RNA was extracted from lung tissue and cultured cells using TRIZol regent (Thermo Fisher Scientific). First-strand cDNA was synthesized from total RNA using ReverTra Ace qPCR RT Master Mix with gDNA Remover (TOYOBO Co., Osaka, Japan). Real-time quantitative PCR analysis was performed using a KAPA SYBR Fast qPCR kit (Kapa Biosystems, Wilmington, MA, USA) with a StepOne system (Thermo Fisher Scientific). Primer sequences are listed in Supplementary Table 1.

### Preparation of LG2055 fraction

LG2055 was suspended in PBS(−) (50 mg/ml) and washed twice with PBS(−) (5,600 × *g* for 15 min at 4°C). Re-suspended LG2055 was sonicated (40 min, on ice). The supernatant was collected (5,600 × *g* for 15 min at 4°C), filtered by 0.22 μm, *in vacuo* condensed, and re-suspended in PBS(−)(water soluble faction). The precipitation was collected, washed twice with PBS(−) and re-suspended in PBS(−) (water insoluble fraction).

### Protein extraction for proteomic analysis

Protein extractions from HEp-2 cells for proteomic experiments were performed using a phase transfer surfactant (PTS) method as described previously^43^. In brief, HEp-2 cells were lysed with a buffer containing 12 mM sodium deoxycholate (SDC), 12 mM sodium *N*-lauroylsarcosinate (SLS) and complete mini protease inhibitors (Roche Diagnostics, Basel, Switzerland) in 100 mM Tris-HCl buffer (pH 9.0) on φ10 cm culture dishes. Cellar proteins were denatured by heating them at 95 °C and sonicated. The soluble protein fraction was collected by centrifugation (20,000 × *g* for 15 min at 4 °C), and protein quantification was performed using a BCA assay (Thermo Fisher Scientific). Aliquots containing 100 μg of proteins were subjected to reductive alkylation, incubated with 10 mM dithiothreitol for 40 min at room temperature, and then 50 mM iodoacetamide was added and incubated for 40 min at r.t. in the dark. After diluting with 50 mM ammonium bicarbonate so that the final concentration of SDC and SLS was 2.4 mM, the extracted proteins were digested with 1 μg of MS grade trypsin (Thermo Fisher Scientific) for 16 h at 37 °C. To remove surfactants from the resultant peptides, an equivalent volume of ethyl acetate was added to the sample solution and trifluoroacetate was further added at a final concentration 0.5% (v/v). The samples were vigorously mixed for 1 min, and the upper layer containing surfactants was removed following centrifugation (20,000 × *g* for 2 min). Finally, the collected water layers were subjected to a styrene divinylbenzene polymer tip column (GL Science, Tokyo, Japan) to desalt.

### MS spectrometry

Extracted peptides were subjected to nanoLC-ESI-MS/MS mass spectrometry (LTQ-LC-MS/MS, Thermo Fisher Scientific) using an EasyNano LC system equipped with a 15-cm ODS column (NTCC-360/75-3-125 C18, particle diameter 3 μm, 0.075 mm × 125 mm; Nikkyo Technos, Tokyo, Japan). The flow rate was adjusted to 300 nL/min for all analyses. The raw data files were processed using software MaxQuant combined with Andromeda (Max Planck Institute of Biochemistry, Bavaria, Germany) for peptide searching, and label-free quantification was carried out using Perseus software (version 1.6.0.7)^44,45^. Peptide precursor mass tolerance was set at 10 ppm, and MS/MS tolerance was set at 0.5 Da. Search criteria included carbamidomethylation of cysteine as a fixed modification and oxidation of methionine (+15.9949) as a variable modification. Searches were performed with full tryptic digestion and a maximum of 2 missed cleavages was allowed. The reverse database search option was enabled, and all peptide data was filtered to satisfy a false discovery rate (FDR) of 1%.

### *In vitro* siRNA experiment

The sequences of sense and antisense siRNAs against SRCAP were, 5′-GAA AAC GAU UGA AGU UGA ATT-3′, 5′-UUC AAC UUC AAU CGU UUC CTT-3′, respectively. Control siRNAs were bought from thermo fisher scientific.

HEp-2 cells were seeded in 6-well dishes at 2.0 × 10^5^ cells/well in 2.0 ml of DMEM supplemented with 10% of FBS. After 24 h incubation at 37 °C, wells were washed three times with PBS, and optiMEM (Thermo fisher scientific) (1.5 ml/well) was added. siRNAs were transfected using lipofectamine RNAiMAX (Thermo fisher scientific) according to manufacturer’s instruction. After 48 h incubation, cells were infected RSV (0.00067 TCID_50_/cell). After 48 h, total RNA was extracted and subjected to quantitative RT-PCR.

### Statistical analysis

Two-tailed Student’s *t* test, Dunnett test and Tukey-kramer test were used for statistical analysis. Statistical analysis of MS data was performed using Perseus (Max Planck Institute of Biochemistry). Statistical analysis for other experiments was performed using R (R Development Core Team, 2015). A *p* value of <0.05 was considered significant. The error bars indicate the standard error of the mean (SEM) or standard deviation (SD).

## Supplementary information

Supplement information

## References

[1] Glezen WP, Taber LH, Frank AL, Kasel JA (1986). Risk of primary infection and reinfection with respiratory syncytial virus. Am. J. Dis. Child..

[2] Nair H (2010). Global burden of acute lower respiratory infections due to respiratory syncytial virus in young children: a systematic review and meta-analysis. Lancet.

[3] Lozano R (2012). Global and regional mortality from 235 causes of death for 20 age groups in 1990 and 2010: a systematic analysis for the Global Burden of Disease Study 2010. Lancet.

[4] Higgins D, Trujillo C, Keech C (2016). Advances in RSV vaccine research and development - A global agenda. Vaccine.

[5] Díez-Domingo J (2014). Social, economic, and health impact of the respiratory syncytial virus: a systematic search. BMC Infect. Dis..

[6] Openshaw PJM, Chiu C, Culley FJ, Johansson C (2017). Protective and harmful immunity to RSV infection. Annu. Rev. Immunol..

[7] Hall CB, Long CE, Schnabel KC (2001). Respiratory syncytial virus infections in previously healthy working adults. Clin. Infect. Dis..

[8] DeVincenzo JP (2015). Activity of oral ALS-008176 in a respiratory syncytial virus challenge study. N. Engl. J. Med..

[9] Reid G (2011). Microbiota restoration: natural and supplemented recovery of human microbial communities. Nat. Rev. Microbiol..

[10] Nakayama Y (2014). Oral administration of *Lactobacillus gasseri* SBT2055 is effective for preventing influenza in mice. Sci. Rep..

[11] Youn HN (2012). Intranasal administration of live *Lactobacillus* species facilitates protection against influenza virus infection in mice. Antiviral Res..

[12] Kawase M, He F, Kubota A, Harata G, Hiramatsu M (2010). Oral administration of lactobacilli from human intestinal tract protects mice against influenza virus infection. Lett. Appl. Microbiol..

[13] Hori T, Kiyoshima J, Shida K, Yasui H (2001). Effect of intranasal administration of *Lactobacillus casei* Shirota on influenza virus infection of upper respiratory tract in mice. Clin. Diagn. Lab. Immunol..

[14] Goto H (2013). Anti-influenza virus effects of both live and non-live *Lactobacillus acidophilus* L-92 accompanied by the activation of innate immunity. Br. J. Nutr..

[15] Weiss G (2010). *Lactobacillus acidophilus* induces virus immune defence genes in murine dendritic cells by a Toll-like receptor-2-dependent mechanism. Immunology.

[16] Fujiwara S, Seto Y, Kimura A, Hashiba H (2001). Establishment of orally-administered *Lactobacillus gasseri* SBT2055SR in the gastrointestinal tract of humans and its influence on intestinal microflora and metabolism. J. Appl. Microbiol..

[17] Takahashi H, Fujita T, Suzuki Y, Benno Y (2006). Monitoring and survival of *Lactobacillus gasseri* SBT2055 in the human intestinal tract. Microbiol. Immunol..

[18] Usman & Hosono, A. Bile tolerance, taurocholate deconjugation, and binding of cholesterol by *Lactobacillus gasseri* strains. *J. Dairy Sci*. **82**, 243–248, 10.3168/jds.S0022-0302(99)75229-X (1999). 10.3168/jds.S0022-0302(99)75229-X 10068945

[19] Sato M (2008). Effects of milk fermented by *Lactobacillus gasseri* SBT2055 on adipocyte size in rats. Br. J. Nutr..

[20] Kadooka Y (2010). Regulation of abdominal adiposity by probiotics (*Lactobacillus gasseri* SBT2055) in adults with obese tendencies in a randomized controlled trial. Eur. J. Clin. Nutr..

[21] Nakagawa H (2016). Effects and mechanisms of prolongevity induced by *Lactobacillus gasseri* SBT2055 in Caenorhabditis elegans. Aging Cell.

[22] Sakai F (2014). *Lactobacillus gasseri* SBT2055 induces TGF-β expression in dendritic cells and activates TLR2 signal to produce IgA in the small intestine. PLoS One.

[23] Shi H (2016). Baicalin from *Scutellaria baicalensis* blocks respiratory syncytial virus (RSV) infection and reduces inflammatory cell infiltration and lung injury in mice. Sci. Rep..

[24] Goritzka M (2014). Alpha/beta interferon receptor signaling amplifies early proinflammatory cytokine production in the lung during respiratory syncytial virus infection. J. Virol..

[25] Nguyen TH (2016). TNF-α and macrophages are critical for respiratory syncytial virus-induced exacerbations in a mouse model of allergic airways disease. J. Immunol..

[26] Borchers AT, Chang C, Gershwin ME, Gershwin LJ (2013). Respiratory syncytial virus—a comprehensive review. Clin. Rev. Allergy Immunol..

[27] González-Sanz R (2016). ISG15 is upregulated in respiratory syncytial virus infection and reduces virus growth through protein ISGylation. J. Virol..

[28] Eichinger KM (2015). Alveolar macrophages support interferon gamma-mediated viral clearance in RSV-infected neonatal mice. Respir. Res..

[29] Behera AK, Kumar M, Lockey RF, Mohapatra SS (2002). 2′-5′ Oligoadenylate synthetase plays a critical role in interferon-gamma inhibition of respiratory syncytial virus infection of human epithelial cells. J. Biol. Chem..

[30] Everitt AR (2013). Defining the range of pathogens susceptible to Ifitm3 restriction using a knockout mouse model. PLoS One.

[31] Goritzka M (2015). Alveolar macrophage-derived type I interferons orchestrate innate immunity to RSV through recruitment of antiviral monocytes. J. Exp. Med..

[32] Todorov SD, Wachsman MB, Knoetze H, Meincken M, Dicks LM (2005). An antibacterial and antiviral peptide produced by *Enterococcus mundtii* ST4V isolated from soya beans. Int J Antimicrob Agents..

[33] Clua P (2017). Peptidoglycan from Immunobiotic *Lactobacillus rhamnosus* improves resistance of infant mice to respiratory syncytial viral infection and secondary pneumococcal pneumonia. Front Immunol..

[34] Iwai A, Takegami T, Shiozaki T, Miyazaki T (2011). Hepatitis C virus NS3 protein can activate the Notch-signaling pathway through binding to a transcription factor, SRCAP. PLoS One.

[35] Ghosh AK (2000). Hepatitis C virus NS5A protein modulates transcription through a novel cellular transcription factor SRCAP. J. Biol. Chem..

[36] Chiba E (2013). Immunobiotic *Lactobacillus rhamnosus* improves resistance of infant mice against respiratory syncytial virus infection. Int. Immunopharmacol..

[37] Kawase M (2012). Heat-killed *Lactobacillus gasseri* TMC0356 protects mice against influenza virus infection by stimulating gut and respiratory immune responses. FEMS Immunol. Med. Microbiol..

[38] Johnston H, Kneer J, Chackalaparampil I, Yaciuk P, Chrivia J (1999). Identification of a novel SNF2/SWI2 protein family member, SRCAP, which interacts with CREB-binding protein. J. Biol. Chem..

[39] Percopo CM (2015). Immunobiotic *Lactobacillus* administered post-exposure averts the lethal sequelae of respiratory virus infection. Antiviral Res..

[40] Fujimura KE (2014). House dust exposure mediates gut microbiome *Lactobacillus* enrichment and airway immune defense against allergens and virus infection. Proc Natl Acad Sci USA.

[41] Wong MM, Cox LK, Chrivia JC (2007). The chromatin remodeling protein, SRCAP, is critical for deposition of the histone variant H2A.Z at promoters. J Biol Chem..

[42] Ye B (2017). Suppression of SRCAP chromatin remodelling complex and restriction of lymphoid lineage commitment by Pcid2. Nat Commun..

[43] Masuda T, Tomita M, Ishihama Y (2008). Phase transfer surfactant-aided trypsin digestion for membrane proteome analysis. J. Proteome Res..

[44] Cox J, Mann M (2008). MaxQuant enables high peptide identification rates, individualized p.p.b.-range mass accuracies and proteome-wide protein quantification. Nat. Biotechnol..

[45] Cox J (2014). Accurate proteome-wide label-free quantification by delayed normalization and maximal peptide ratio extraction, termed MaxLFQ. Mol. Cell. Proteomics.

