# Supplementary material for: Prevention of respiratory syncytial virus infection with probiotic lactic acid bacterium Lactobacillus gasseri SBT2055
Source: Sci Rep. 2019 Mar 18;9:4812. doi: 10.1038/s41598-019-39602-7 (PMC6423325; doi:10.1038/s41598-019-39602-7)
Supplement: Supplementary file 1 — Supplement information [file 41598_2019_39602_MOESM1_ESM.pdf]

Supplementary Information

Prevention of respiratory syncytial virus infection with probiotic lactic acid bacterium *Lactobacillus gasseri* SBT2055

Kei Eguchi[1#], Naoki Fujitani[2#], Hisako Nakagawa[2], and Tadaaki Miyazaki[2*]

[1]Milk Science Research Institute, Megmilk Snow Brand Co., Ltd., 1-2, Minamidai, 1-chome, Kawagoe, Saitama 350-1165, Japan

[2]Department of Probiotics Immunology, Institute for Genetic Medicine, Hokkaido University, N15W7, Kita-ku, Sapporo 060-0815, Japan

[#]Contributed equally to this work

*To whom correspondence should be addressed:
Tadaaki Miyazaki, Ph.D., Department of Probiotics Immunology, Institute for Genetic Medicine Hokkaido University, N15, W7 Kita-ku, Sapporo, Japan.
E-mail: miyazaki@igm.hokudai.ac.jp
Phone and fax: +81-11-706-9085

Table S1 qPCR primer list used in this study

gene name	sense	antisense
RSV-F	GGAACAAGTTGTTGAGGTTTATGAATATGC	TTCTGCTGTCAAGTCTAGTACACTGTAGT
Rpl19	ATGAGTATGCTCAGGCTACAGA	GCATTGGCGATTTCATTGGTC
HIST1H4	TCCTGGAGAATGTGATACGG	TTGAGCGCGTAGACAACATC
IFN-β	TCCAGCACTGGGTGGAATGA	GGTACCTTTGCACCCTCCAG
IFN-γ	CTGCAGAGCCAGATTATCTC	CCTGTGGGTTGTTGACCTCA
Oas1a	GAAGAGGCTGATGTGTGGCT	TGTCCAGTTCTCTTCTACCTGC
Mx1	CCAACTGGAATCCTCCTGGAA	GCCGCACCTTCTCCTCATAG
ISG15	GAGCTAGAGCCTGCAGCAAT	TTCTGGGCAATCTGCTTCTT
SRCAP	CTCCACTGCTACCTCGTTTGGT	GGAAAATCCGTTCCAGGCGTTC
GAPDH	GTCTCCTCTGACTTCAACAGCG	ACCACCCTGTTGCTGTAGCCAA

Table S2 up- and down-regulated proteins by addition of LG2055 water-soluble fraction

**Protein names**	**Gene names**	**Log2-fol**
E3 ubiquitin-protein ligase MYCBP2	MYCBP2	-4.504
Helicase SRCAP	SRCAP	-4.487
Death-associated protein kinase 2;Death-associated protein kinase 3	DAPK2;DAPK3	-4.101
POU domain, class 3, transcription factor 2;POU domain, class 3, transcription factor 1;POU domain, class 3, transcr	POU3F2;POU3F1;POU3F3	-3.878
60S ribosomal protein L15	RPL15	-3.808
Histone H3.2	HIST2H3A	-3.304
Heterogeneous nuclear ribonucleoprotein U-like protein 2	HNRNPUL2	-3.187
Tropomyosin alpha-3 chain	TPM3	-3.130
3-beta-hydroxysteroid-Delta(8),Delta(7)-isomerase	EBP	-3.096
Derlin-2	DERL2	-3.091
Structural maintenance of chromosomes flexible hinge domain-containing protein 1	SMCHD1	-2.999
Histone H1x	H1FX	-2.955
ATP synthase subunit gamma, mitochondrial	ATP5C1	-2.891
BTB/POZ domain-containing protein KCTD16	KCTD16	-2.852
N-terminal Xaa-Pro-Lys N-methyltransferase 1;N-terminal Xaa-Pro-Lys N-methyltransferase 1, N-terminally processed	NTMT1	-2.787
Fibroblast growth factor 8	FGF8	-2.711
Transmembrane channel-like protein 1	TMC1	-2.687
Protein NipSnap homolog 1	NIPSNAP1	-2.626
Syntaxin-7	STX7	-2.583
Helicase POLQ-like	HELQ	-2.487
Catenin delta-1	CTNND1	-2.476
ADP/ATP translocase 1	SLC25A4	-2.343
UDP-glucose 6-dehydrogenase	UGDH	-2.310
Splicing factor 3A subunit 3	SF3A3	-2.264
Putative uncharacterized protein LRRC75A-AS1, mitochondrial	LRRC75A-AS1	-2.161
Protein mago nashi homolog;Protein mago nashi homolog 2	MAGOH;MAGOHB	-2.144
Splicing factor U2AF 26 kDa subunit;Splicing factor U2AF 35 kDa subunit	U2AF1L4;U2AF1	-2.118
Major vault protein	MVP	-2.077
Coatomer subunit gamma-1	COPG1	-2.032

**Protein names**	**Gene names**	**Log2-fol**
Protein monoglycylase TTLL8	TTLL8	4.066
Transcription elongation factor A N-terminal and central domain-containing protein 2	TCEANC2	3.692
Inorganic pyrophosphatase	PPA1	3.235
Proline-rich basic protein 1	PROB1	3.230
Histone-lysine N-methyltransferase SUV420H1	SUV420H1	3.132
LIM domain and actin-binding protein 1	LIMA1	3.097
Retinoic acid receptor beta	RARB	2.826
Cystathionine beta-synthase	CBS	2.776
ATP-dependent RNA helicase DDX19A;ATP-dependent RNA helicase DDX19B	DDX19A;DDX19B	2.672
AP-2 complex subunit beta	AP2B1	2.548
Isopentenyl-diphosphate Delta-isomerase 1	IDI1	2.476
Eukaryotic translation initiation factor 3 subunit M	EIF3M	2.471
Zinc finger protein 728	ZNF728	2.444
MICOS complex subunit MIC19	CHCHD3	2.286
Phosphatidylinositol 5-phosphate 4-kinase type-2 alpha	PIP4K2A	2.192
Prostaglandin G/H synthase 2	PTGS2	2.182
Transmembrane protein 2	TMEM2	2.131
Dynein heavy chain 2, axonemal	DNAH2	2.065
Serine--tRNA ligase, cytoplasmic	SARS	2.020

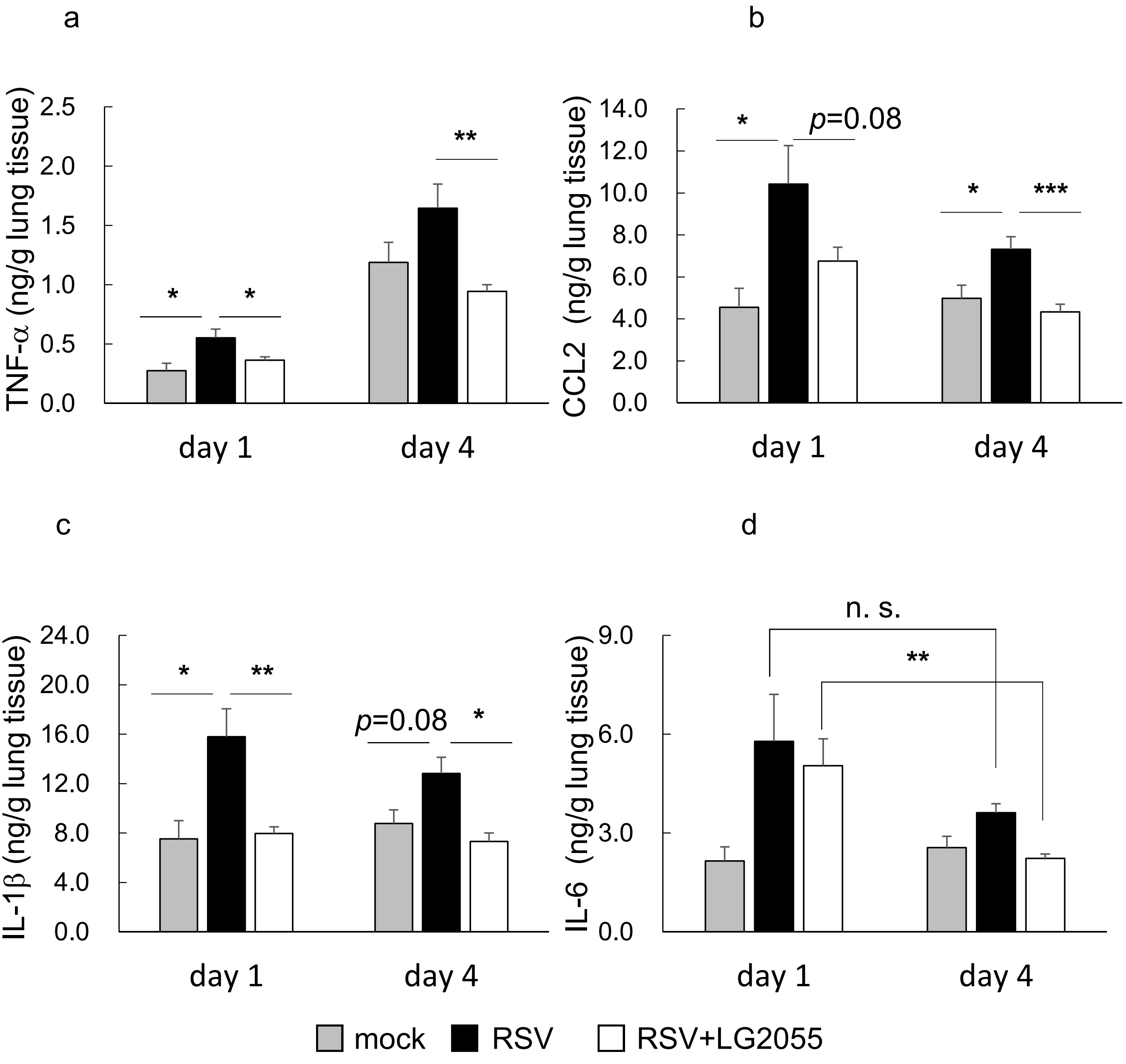

Supplement Figure 1: Orally administered LG2055 reduced lung proinflammatory cytokines production at day1 and day4 postinfection with RSV.

Mice were orally administered LG2055 (2 × 10^9 cfu/head/day) or 25(%) trehalose solution for 21 d, and the mice were infected RSV or not infected. day1, mock: n=5, hRSV: n=9, RSV+LG2055: n=9, and day4, mock: n=5, RSV: n=10, RSV+LG2055: n=10. (a) TNF-α, (b) CCL2, (c) IL-1β, (d) IL-6 concentration in lung homogenates were measured using ELISA. Data are presented as means + SEM. * $p < 0.05$, ** $p < 0.01$ and *** $p < 0.001$ (student's *t* test).

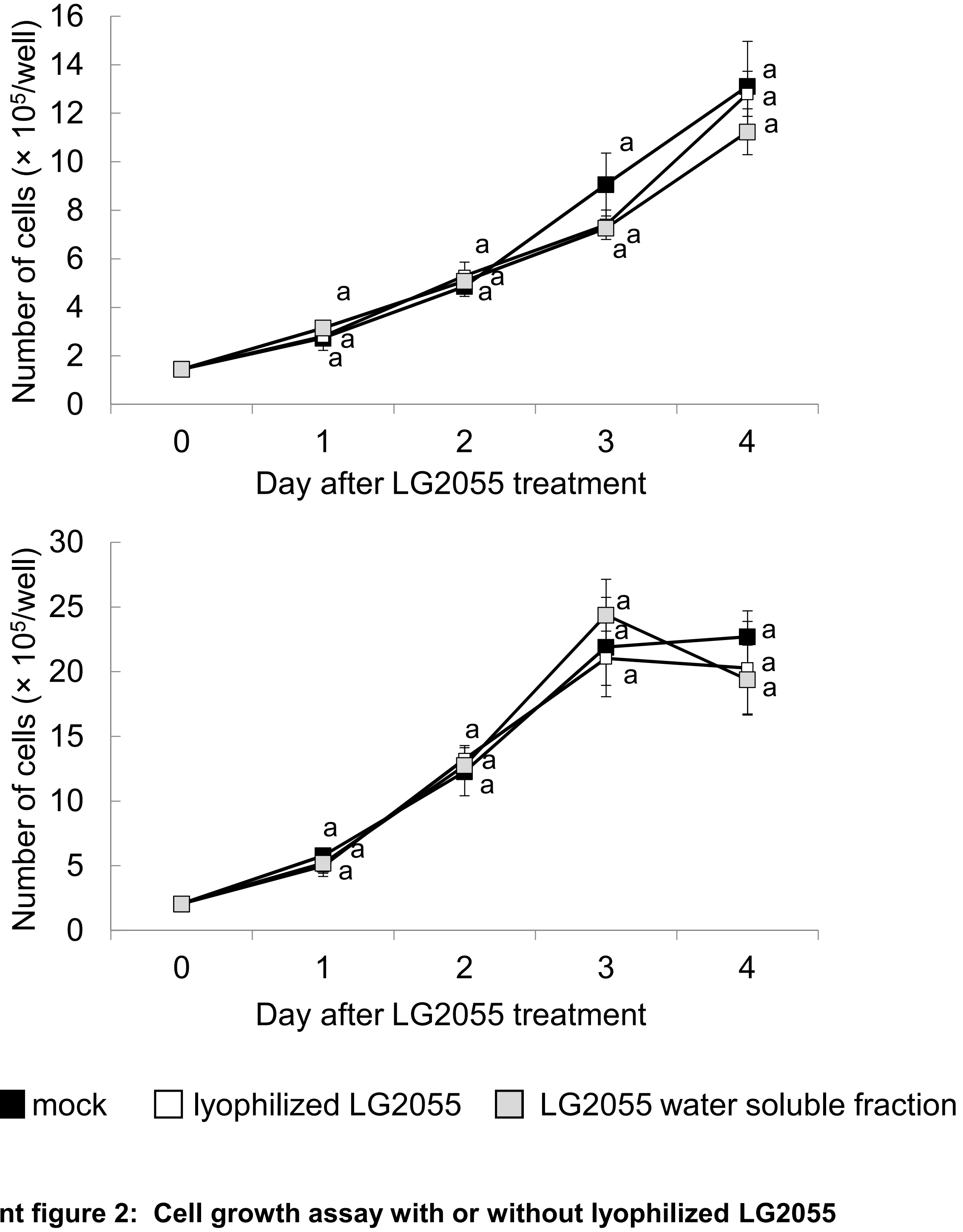

Supplement figure 2: Cell growth assay with or without lyophilized LG2055
Cells were prepared in 24-well dishes and treated with LG2055 or its soluble fraction. After 1,2,3 or 4 days incubation, each well was treated with Trypsin-EDTA (0.25%), phenol red (Thermo fisher Scientific) and the number of total cells were measured. (a) HEp-2 cell, (b) MLE12 cell. Data are presented as means±SD. Means with the same letter do not differ ($P \geq 0.05$, Tukey-kramer test).

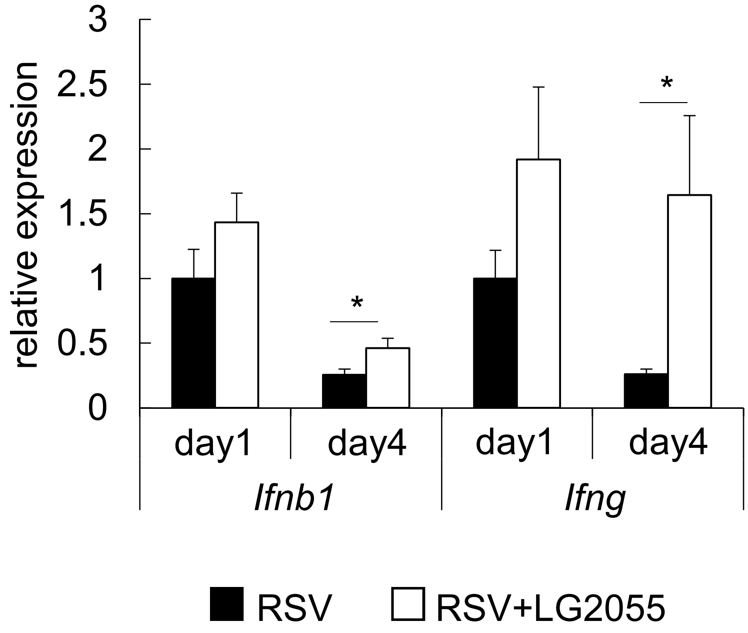

Supplement Figure 3: IFN gene expression level in mouse lung at day 1 postinfection. Mice were orally administered LG2055 (2 × 109 cfu/head/day) or a 25% trehalose solution for 21 d, and were then infected with RSV or not infected (mock: n=4, RSV: n=8 and RSV+LG2055: n=10). Gene expression in lung homogenates at day 1 postinfection was assessed by real-time PCR analysis. Data are presented as means + SEM. * $p < 0.05$, ** $p < 0.01$ and *** $p < 0.001$ (student's *t* test).

Identities 9/12 (75%)
Positives 10/12 (83%)

```
 HCV NS3   14 LGTIVTSLTGRD 25
              L TI+TSLT RD
hRSV NS2   23 LETIITSLT-RD 33
```

Supplement Figure 4: Result of alignment between HCV NS3 and hRSV NS2 by BLAST search

Sequence homology between HCV and NS proteins of RSV was investigated by BLAST search. Sequence alignment between HCV NS3 and RSV NS2 was also assessed by BLAST search.
